# TAT-ODD-p53 enhances the radiosensitivity of hypoxic breast cancer cells by inhibiting Parkin-mediated mitophagy

**DOI:** 10.18632/oncotarget.4002

**Published:** 2015-05-18

**Authors:** Rong Zheng, Qiwei Yao, Guozhu Xie, Shasha Du, Chen Ren, Yuxia Wang, Yawei Yuan

**Affiliations:** ^1^ Department of Radiation Oncology, Nanfang Hospital, Southern Medical University, Guangzhou, Guangdong 510515, P.R. China; ^2^ Beijing Institute of Pharmacology and Toxicology, Beijing 100850, China

**Keywords:** p53, mitophagy, radiosensitivity, Parkin

## Abstract

Radiation therapy has an important role in the treatment of breast cancer. Dysfunction p53 and hypoxia are typical biological characteristics of breast cancer that constitute barriers to the efficacy of radiotherapy. Mitophagy plays a protective role in cellular homeostasis under hypoxic conditions, while mitophagy is inhibited by p53 in normal cells. We explored the effects of a p53 fusion protein, TAT-ODD-p53, on the radiosensitivity of hypoxic breast cancer cells both *in vitro* and *in vivo*, as well as investigating the related molecular mechanisms. We found that selective accumulation of TAT-ODD-p53 occurred under hypoxic conditions and significantly increased tumor cell radiosensitivity both *in vitro* and *in viv*o. Mitophagy had an important role in maintaining hypoxia-induced radioresistance. Mitophagy was inhibited by TAT-ODD-p53 and this inhibition was suppressed by over-expression of Parkin in hypoxic irradiated breast cancer cells. In addition, mitophagy was induced by deletion of p53, with this effect being weakened by Parkin knockdown at a low oxygen tension. By interacting with Parkin, p53 inhibited the translocation of Parkin to the mitochondria, disrupting the protective mitophagy process. These results suggest that TAT-ODD-p53 has a significant and preferential radiosensitizing effect on hypoxic breast cancer cells by inhibition of Parkin-mediated mitophagy.

## INTRODUCTION

Among cancers affecting in women, breast cancer has the highest incidence and causes the second highest mortality [1]. Current treatment for breast cancer often involves radiation therapy, which is widely used and effective. However, hypoxic tumor cells usually exhibit radioresistance, which poses a challenge for successful radiation therapy [2]. Hypoxic regions are found in most solid tumors, but seldom occur in normal tissues [3, 4]. Therefore, hypoxic cells are an attractive tumor-specific target for improving the response to ionizing radiation.

It has been reported that tumor cells expressing wild-type p53 are more sensitive to radiation than p53 knockout cells in the presence of hypoxia [5], suggesting that a hypoxic environment may select for cells with loss of p53 function [6]. In some tumor cell lines, hypoxia may decrease radiosensitivity by suppression of p53 activity [7], while reactivation of p53 seems to be an effective method of targeting hypoxic tumors [8]. Because hypoxic regions are uncommon in normal tissues, synthetic p53 peptides specifically targeting hypoxic cancer cells could provide a novel approach to radiosensitization.

Mitophagy is a type of autophagy that occurs in the mitochondria, and it plays a critical role in the selective removal of damaged or unwanted mitochondria to avoid cell death [9]. The protein Parkin is selectively recruited to dysfunctional mitochondria with a low membrane potential, where it subsequently mediates the degradation of impaired mitochondria by autophagosomes [10]. It has been found that Parkin-mediated mitophagy is also induced by hypoxia, and helps to restore cellular homeostasis by degrading defective mitochondria [11]. Since autophagy has been reported to be involved in tumor resistance to radiotherapy [12], we hypothesized that Parkin-mediated mitophagy might contribute to hypoxic radioresistance.

While p53 regulates autophagy as a nuclear transcription factor [13], it also inhibits the process of autophagy by a poorly characterized extranuclear mechanism [14]. Moreover, there is increasing evidence that p53 inhibits mitophagy in normal cells [15, 16]. Consistent with such findings, it has been reported that cytosolic p53 can also disturb the process of mitophagy by inhibiting Parkin in mouse heart cells and pancreatic β-cells [17, 18]. Despite these recent reports about regulation of mitophagy by p53 in normal cells, the role of p53 in tumor cell mitophagy remains poorly understood.

We previously constructed a novel fusion protein consisting of wild-type p53 combined with TAT domain_47–57_ and the minimum oxygen-dependent degradation domain_557–574_ (ODD) of hypoxia-induced factor-1 α (HIF-1α). We found that TAT-ODD-p53 (TOP) was successfully delivered into cells via the TAT protein transduction domain and selectively stabilized in hypoxic tumor tissues under regulation of the ODD domain [19, 20]. In this study, we evaluated the use of TAT-ODD-p53 for targeted radiosensitization both *in vitro* and *in vivo* and investigated the underlying mechanisms. Our findings suggested that TAT-ODD-p53 has a significant radiosensitizing effect on hypoxic breast cancer cells that is due to inhibition of Parkin-mediated mitophagy.

## RESULTS

### Transmembrane delivery, location, and stability of synthetic p53 peptides

**Figure 1:**
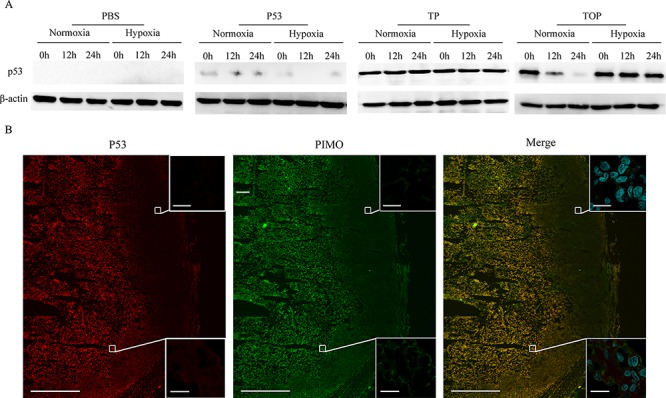
Cell permeability and selective stability of p53 fusion proteins in the MDA-MB-157 cell line and in tumor tissue **A.** Western blotting was used to assess the stability of p53, TAT-p53 (TP), and TAT-ODD-p53 (TOP) at different times under different oxygenation conditions. **B.** Co-localization of TOP and pimonidazole (PIMO) in tumor tissue was detected by immunofluorescence using confocal microscopy (pimonidazole-positive viable fraction is green; TOP is red). (Scale bars = 1000 μm). Magnified photographs show an overlapping region (lower box) and a non-overlapping region (upper box) (Scale bar = 20 μm).

To assess intracellular delivery of the synthetic p53 peptides, MDA-MB-157 cells (null p53) were treated with PBS or p53 peptides at a final concentration of 10 μg/ml for 1 h under normoxic (20% O_2_) or hypoxic (0.5% O_2_) conditions. Western blotting showed that expression of p53 was only detectable in cells treated with the TAT-p53 or TAT-ODD-p53 fusion proteins, suggesting that p53 fusion proteins conjugated with TAT could be effectively delivered intracellularly *in vitro*. Importantly, p53 was delivered stably under both normoxic and hypoxic conditions when the cells were treated with TAT-p53. In contrast, p53 was rapidly degraded under normoxic conditions when cells were treated with TAT-ODD-p53, but it remained stable under hypoxic conditions (Figure 1A). These results demonstrated that TAT-ODD-p53 penetrated the cells sufficiently and was selectively stable under hypoxic conditions.

To assess whether TAT-ODD-p53 showed selective accumulation in the hypoxic regions of breast tumors *in vivo*, we used immunofluorescence staining to analyze the co-localization of TAT-ODD-p53 and pimonidazole, which is a probe that selectively stains hypoxic tissue. We found that TAT-ODD-p53 was localized with pimonidazole in similar areas of tumor tissue, indicating that TAT-ODD-p53 showed preferential accumulation and remained stable in the hypoxic regions of tumor tissue (Figure 1B).

### Radiosensitization of breast cancer cells by synthetic p53 peptides *in vitro*

**Figure 2:**
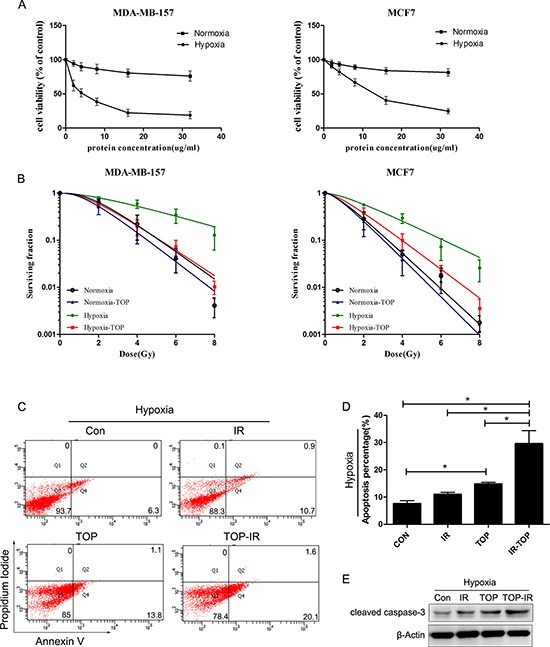
TOP inhibits tumor cell growth and enhances radiosensitivity under normoxic and hypoxic conditions *in vitro* **A.** Inhibition of cell growth by p53 fusion proteins under different oxygenation conditions (hypoxia, 0.5% O_2_; normoxia, 20% O_2_) was assessed by the MTT assay (mean ± SD, *n* = 3). **B.** Clonogenicity survival assay of MDA-MB-157 cells and MCF7 cells exposed to TOP and 0–8 Gy of radiation under different oxygenation conditions (hypoxia, 0.5% O_2_; normoxia, 20% O_2_) (mean ± SD, *n* = 3). **C-E.** Apoptosis of hypoxic MDA-MB-157 cells exposed to TOP or/and irradiation (IR) was measured by externalization of Annexin V (flow cytometry) and cleaved caspase-3 (western blotting) (mean ± SD, *n* = 3; *P < 0.05).

To choose a suitable dosage for further investigation, we analyzed the inhibition of cell growth by p53 fusion proteins in different O_2_ environments. Breast cancer cells were incubated with various concentrations of p53 fusion proteins (0–32 μg/ml) for 72 h under hypoxic (0.5% O_2_) or normoxic (20% O_2_) conditions and cell viability was assessed with the MTT assay (Figure 2A). TAT-ODD-p53 only induced significant reduction of cell viability under low oxygen conditions, suggesting that TAT-ODD-p53 could selectively inhibit tumor cell growth in hypoxic regions. The IC_50_ value [21] of TAT-ODD-p53 for breast cancer cells under low oxygen conditions was calculated to be 4.00 μg/ml and 12.85 μg/ml for MDA-MB-157 cells (null p53) and MCF-7 cells (wild-type p53), respectively. Subsequent experiments were carried out using a concentration of 4 μg/ml and these two cell lines.

To determine the radio-sensitizing effect of TAT-ODD-p53, a colony-forming assay was performed using MCF-7 cells and MDA-MB-157 cells exposed to radiation after incubation for 1 h with TAT-ODD-p53 under normoxic or hypoxic conditions. The radioprotective effect of hypoxia can be expressed quantitatively by calculating the oxygen enhancement ratio (OER) [22]. The OER of MCF-7cells and MDA-MB-157 cells was 1.91 and 2.43, respectively, suggesting that hypoxia induced significant radioresistance. The sensitizer enhancement ratio (SER) was calculated as the radiation dose that resulted in a surviving fraction of 37% (D0 in radiobiology) divided by the dose needed for the same surviving fraction when cells were exposed to TAT-ODD-p53 plus radiation. TAT-ODD-p53 sensitized MCF-7 cells and MDA-MB-157 cells to ionizing radiation, with SERs of up to 1.55 and 2.24, respectively (Figure 2B).

Apoptosis was measured by flow cytometry (FCM) and western blotting in MDA-MB-157 cells treated with TAT-ODD-p53 (4 μg/ml) under hypoxic conditions at 48 h after irradiation (6 Gy). As shown in Figure 2C and 2D, radiation-induced apoptosis increased notably under hypoxic conditions after exposure to TAT-ODD-p53. There was a consistent and significant increase of caspase-3 cleavage in the combination group under hypoxic conditions (Figure 2E). Taken together, these results provided further evidence that TAT-ODD-p53 enhanced the radiosensitivity of hypoxic breast cancer cells.

### Radiosensitization of breast cancer cells by synthetic p53 peptides *in vivo*

**Figure 3:**
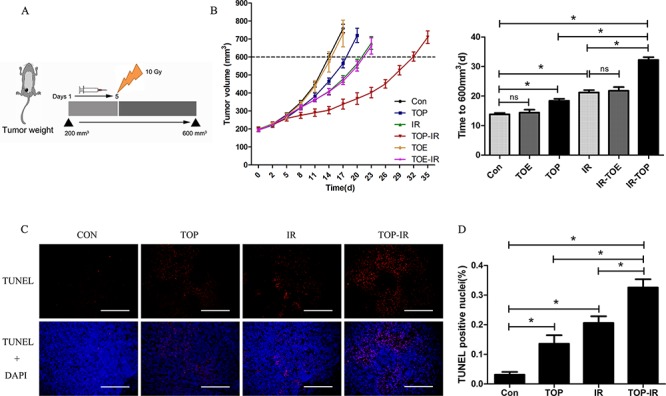
TOP inhibits tumor growth and enhances radiosensitivity *in vivo* **A.** When tumors reached a volume of approximately 200 mm^3^, the mice were treated with PBS (Con), TAT-ODD-EGFP (TOE), or TOP for 5 days and received 10 Gy of radiation on day 5 (mean ± SD, *n* = 5). **B.** Pretreatment with TOP resulted in supra-additive tumor growth delay. Columns show the mean time for the tumors to reach 600 mm^3^ in each treatment group (mean ± SD, *n* = 5; *P < 0.05). **C.** Frozen tumor tissue sections were subjected to the TUNEL assay and counterstained with DAPI. (Scale bars = 500 μm.) **D.** The mean percentage of TUNEL-positive cells was counted in three sections from three tumor tissue samples in each experimental group (mean ± SD, *n* = 3; *P < 0.05).

The *in vivo* effects of TAT-ODD-p53 were evaluated in combination with local tumor irradiation. Nude mice with subcutaneous MDA-MB-157 xenograft tumors were treated with TAT-ODD-p53 (1 mg/kg, i.p.) every day for five days before a single 10 Gy dose of radiation and tumor growth was monitored until the maximum permitted volume (600 mm^3^) was reached. In unirradiated mice treated with PBS or TAT-ODD-p53, the tumors reached this volume on day 14 and 18, respectively. However, the time to reach the maximum permitted volume was 21 days in mice receiving radiation alone and 32 days in mice treated by radiation combined with TAT-ODD-p53, demonstrating distinct supra-additive tumor growth delay. In contrast, TAT-ODD-EGFP did not inhibit the growth or enhance the radiosensitivity of MDA-MB-157 xenografts established in nude mice, demonstrating that the TAT-ODD domain had no antitumor activity (Figure 3A, 3B).

DNA fragmentation was detected by the TdT-mediated dUTP nick end labeling (TUNEL) assay, and DAPI was used as a nuclear marker. It was found that TAT-ODD-p53 significantly increased the number of apoptotic nuclei with fragmented DNA in tumor tissues at seven days after irradiation (Figure 3C, 3D). Consistent with previous data, the TUNEL assay showed a significant increase in the number of apoptotic cells in the combined treatment group compared with the TAT-ODD-p53 alone or irradiation alone groups.

### TAT-ODD-p53 may sensitize hypoxic cells to irradiation by inhibition of mitophagy

**Figure 4:**
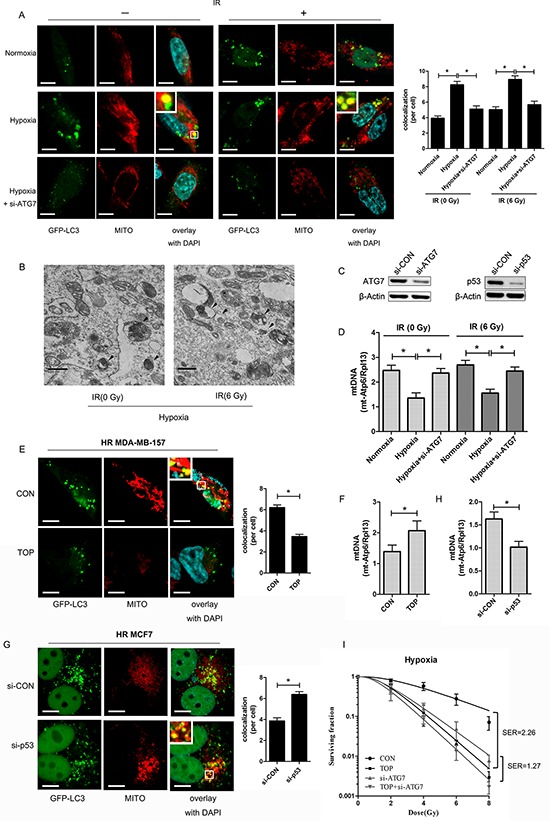
TOP inhibits hypoxia-induced protective mitophagy in breast cancer cells exposed to irradiation **A.** Representative images of GFP-LC3-overexpressing MDA-MB-157 cells exposed to hypoxia and ATG7 siRNA with or without irradiation (6 Gy). Mitochondria are stained red with MitoTracker (Mito). At least 30 GFP-positive cells were scored in three independent experiments to assess the co-localization of LC3 and mitochondria (mean ± SD, *n* = 3; *P < 0.05). These analyses were performed using confocal microscopy (scale bar = 10 μm). **B.** Representative electron micrographs of MDA-MB-157 cells exposed to hypoxia with or without irradiation (6 Gy). Arrows indicate an autophagic vacuole containing mitochondria (original magnification × 30, 000). (Scale bars = 600 nm). **C.** Expression of ATG7 in MDA-MB-157 cells transfected with ATG7 siRNA and p53 expression in MCF7 cells transfected with p53 siRNA detected by western blotting. **D.** Expression of mt-Atp6 and Rpl13 in MDA-MB-157 cells exposed to hypoxia and ATG7 siRNA with or without irradiation (6 Gy) was assessed by real-time PCR. Relative mitochondrial DNA levels are indicated as mt-Atp6/Rpl13 (mean ± SD, *n* = 6; *P < 0.05). **E.** Representative images of GFP-LC3-overexpressing hypoxic irradiated (0.5% O_2_, 6 Gy) (HR) MDA-MB-157 cells treated with TOP. The mitochondria were stained red with MitoTracker (Mito). At least 30 GFP-positive cells were scored in three independent experiments to measure the co-localization of LC3 and mitochondria (mean ± SD, *n* = 3; *P < 0.05). These analyses were performed using confocal microscopy (scale bar = 10 μm). **F.** Expression of mt-Atp6 and Rpl13 in HR MDA-MB-157 cells treated with TOP was assessed by real-time PCR. Relative mitochondrial DNA levels are indicated as mt-Atp6/Rpl13 (mean ± SD, *n* = 6; *P < 0.05). **G.** Representative images of GFP-LC3-overexpressing HR MCF7 cells transfected with p53 siRNA. Mitochondria were stained red with MitoTracker (Mito). At least 30 GFP-positive cells were scored in three independent experiments to measure the co-localization of LC3 and mitochondria (mean ± SD, *n* = 3; *P < 0.05). These analyses were performed using confocal microscopy (scale bar = 10 μm). **H.** Expression of mt-Atp6 and Rpl13 in HR MCF7 cells transfected with p53 siRNA was assessed by real-time PCR. Relative mitochondrial DNA levels are indicated as mt-Atp6/Rpl13 (mean ± SD, *n* = 6; *P < 0.05). **I.** Clonogenicity survival assay of hypoxic MDA-MB-157 cells transfected with ATG7 siRNA and/or TOP, followed by exposure to 0–8 Gy of radiation (mean ± SD, *n* = 3).

Clearance of defective mitochondria by an autophagic process is termed mitophagy and is essential for mitochondrial homeostasis. Co-localization of LC3 (GFP-LC3; green fluorescence), a marker of autophagosomes [23] and mitochondria (MitoTracker; red fluorescence) was evaluated for detection of mitophagy. Quantification of co-localization showed that more mitochondria and autophagosomes were co-localized in hypoxic cells with or without irradiation, suggesting that mitophagy was induced under low oxygen conditions (Figure 4A). Electron microscopy revealed that mitochondria were incorporated into autophagic vacuoles in the hypoxic cells (Figure 4B), providing further evidence that mitophagy was induced in breast cancer cells by hypoxia.

In addition, real-time PCR was employed to determine the expression of mt-Atp6 (mitochondrial DNA; mtDNA) and Rpl13 (genomic DNA), since the ratio reflects the relative number of mitochondria per cell [24]. It was found that the mtDNA content decreased in irradiated cells exposed to hypoxia, while this change was reversed by Atg7 knockdown [23] (Figure 4C, 4D), suggesting that mitophagy participated in mitochondrial clearance under hypoxic conditions. Immunofluorescence of hypoxic irradiated MDA-MB-157 cells demonstrated a decrease of co-localization between LC3 and mitochondria (Figure 4E), while the number of mitochondria was increased in TOP group (Figure 4F). Mitophagy was also enhanced in hypoxic irradiated MCF7 cells (wild-type p53) after transfection with siRNA for p53 (Figure 4G), while the number of mitochondria decreased after p53 knockdown (Figure 4H). These results suggested that TAT-ODD-p53 inhibited hypoxia-induced mitophagy in breast cancer cells.

To further clarify whether an effect on mitophagy was involved in radio-sensitization of hypoxic cells by TAT-ODD-p53, we employed the colony-forming assay to evaluate the radiosensitivity of hypoxic MDA-MB-157 cells treated with TAT-ODD-p53 and/or Atg7 siRNA [24]. Hypoxic cells were more sensitive to radiation when mitophagy was inhibited by Atg7 knockdown, with an SER of 1.92 (Figure 4I). The radiosensitizing effect of TAT-ODD-p53 was attenuated after autophagy was blocked by Atg7 knockdown, since the SER of TAT-ODD-p53 for hypoxic cells transfected with Atg7 (1.27) was lower than that for the control group (2.26) (Figure 4I), suggesting TAT-ODD-p53 increases the sensitivity of hypoxic cells to irradiation at least partly through inhibition of mitophagy.

### TAT-ODD-p53 inhibits mitophagy in hypoxic cells by suppressing translocation of Parkin to the mitochondria

**Figure 5:**
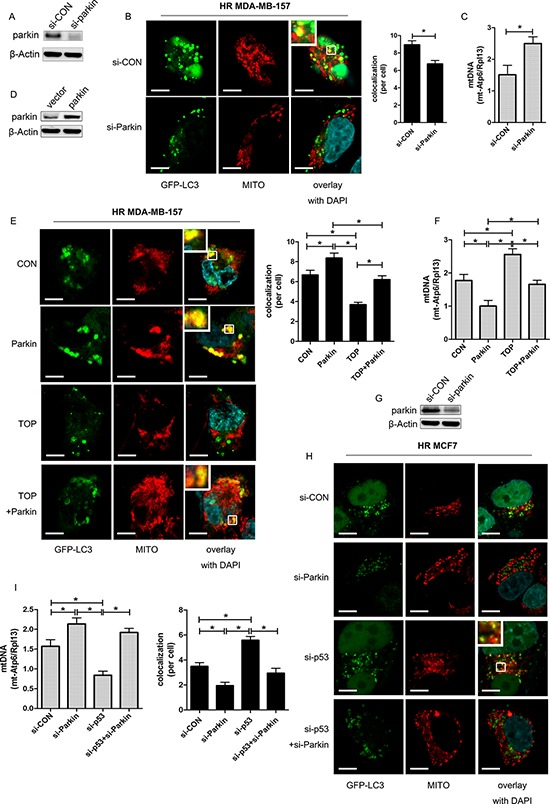
TOP inhibits Parkin-mediated mitophagy under hypoxic conditions **A.** Expression of Parkin in MDA-MB-157 cells transfected with siRNA targeting Parkin (si-Parkin) was detected by western blotting. **B.** Representative images of GFP-LC3-overexpressing HR MDA-MB-157 cells transfected with si-Parkin. Mitochondria were stained red with MitoTracker (Mito). At least 30 GFP-positive cells were scored in three independent experiments to measure the co-localization of LC3 and mitochondria (mean ± SD, *n* = 3; *P < 0.05). These analyses were performed using confocal microscopy (scale bar = 10 μm). **C.** Expression of mt-Atp6 and Rpl13 in HR MDA-MB-157 cells transfected with si-Parkin was assessed by real-time PCR. Relative mitochondrial DNA levels are indicated as mt-Atp6/Rpl13 (mean ± SD, *n* = 6; *P < 0.05). **D.** Expression of Parkin in MDA-MB-157 cells transfected with Parkin plasmid was detected by western blotting. **E.** Representative images of GFP-LC3-overexpressing HR MDA-MB-157 cells transfected with Parkin plasmid and/or TOP. Mitochondria were stained red with MitoTracker (Mito). At least 30 GFP-positive cells were scored in three independent experiments to measure the co-localization of LC3 and mitochondria (mean ± SD, *n* = 3; *P < 0.05). These analyses were performed using confocal microscopy (scale bar = 10 μm). **F.** Expression of mt-Atp6 and Rpl13 in HR MDA-MB-157 cells transfected with Parkin plasmid and/or TOP was assessed by real-time PCR. Relative mitochondrial DNA levels are indicated as mt-Atp6/Rpl13 (mean ± SD, *n* = 6; *P < 0.05). **G.** Expression of p53 in MCF7 cells transfected with p53 siRNA was detected by western blotting. **H.** Representative images of GFP-LC3-overexpressing HR MCF7 cells transfected with p53 siRNA and/or Parkin siRNA. Mitochondria were stained red with MitoTracker (Mito). At least 30 GFP-positive cells were scored in three independent experiments to measure the co-localization of LC3 and mitochondria (mean ± SD, *n* = 3; *P < 0.05). These analyses were performed using confocal microscopy (scale bar = 10 μm). **I.** Expression of mt-Atp6 and Rpl13 in HR MCF7 cells transfected with p53 siRNA or/and Parkin siRNA was assessed by real-time PCR. Relative mitochondrial DNA levels are indicated as mt-Atp6/Rpl13(mean ± SD, *n* = 6; *P < 0.05).

Parkin is an E3 ubiquitin ligase that selectively undergoes translocation into damaged mitochondria to initiate mitophagy [25]. In irradiated MDA-MB-157 cells, we found that hypoxia-induced mitophagy was inhibited by Parkin knockdown (Figure 5A, 5B). The mtDNA content was elevated in hypoxic cells with depletion of Parkin, suggesting that clearance of mitochondria by mitophagy was reduced (Figure 5C). These results indicated that mitophagy might occur via the Parkin-dependent pathway under hypoxic conditions.

Overexpression of Parkin partly reversed the inhibitory effect of TAT-ODD-p53 on mitophagy in MDA-MB-157 cells (Figure 5D, 5E), and the number of mitochondria was also increased in the combination group compared with the TOP group (Figure 5F). In addition, p53 knockdown-mediated induction of co-localization between LC3 and mitochondria in MCF7 cells under hypoxic conditions was eliminated by deletion of Parkin (Figure 5G, 5H), and the number of mitochondria was no longer decreased by p53 knockdown after inhibition of Parkin (Figure 5I). These results demonstrated that TAT-ODD-p53 blocked mitophagy via the Parkin-dependent pathway under hypoxic conditions.

**Figure 6:**
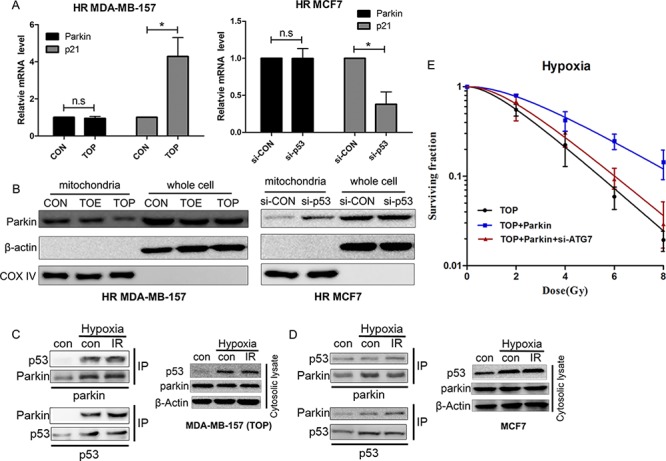
Interaction of p53 with Parkin inhibits translocation of Parkin to the mitochondria and suppresses hypoxia-induced radioresistance via inhibition of mitophagy **A.** Relative expression of Parkin mRNA in HR MDA-MB-157 cells treated with TOE/TOP or HR MCF7 cells transfected with p53 siRNA. p21 was used as a positive control. **B.** Parkin expression in mitochondria or whole cell extracts by western blotting. **C.** The Parkin-p53 complex in MDA-MB-157 cells. The cytosolic lysate of MDA-MB-157 cells treated with TOP and exposed to radiation (6 Gy) under low oxygen conditions (0.5% O_2_) was subjected to immunoprecipitation with anti-p53 and anti-Parkin antibodies. **D.** The Parkin-p53 complex in MCF7 cells. The cytosolic lysate of MCF7 cells exposed to radiation (6 Gy) under low oxygen conditions (0.5% O_2_) was subjected to immunoprecipitation with anti-p53 and anti-Parkin antibodies. **E.** Clonogenicity survival assay of hypoxic MDA-MB-157 cells transfected with TOP, Parkin, or ATG7 siRNA and exposed to 0–8 Gy of radiation (mean ± SD, *n* = 3).

To further confirm the mechanism by which p53 acted on Parkin-mediated mitophagy, expression of Parkin was tested by qRT-PCR and western blotting (Figure 6A, 6B). P53 did not alter Parkin expression in hypoxic breast cancer cells, but it affected translocation of Parkin to the mitochondria. In MDA-MB-157 cells, mitochondrial translocation of Parkin was inhibited by TAT-ODD-p53. Since translocation was increased by p53 knockdown in MCF7 cells, TAT-ODD itself did not regulate the translocation of Parkin. Because transcription-independent modulation of autophagy by cytoplasmic p53 has often been reported [14] and Parkin undergoes selective translocation to impaired mitochondria to initiate mitophagy [9], the interaction between p53 and Parkin proteins was examined in the cytosolic lysate of MDA-MB-157 cells. After treatment with TAT-ODD-p53, the Parkin-p53 complex was observed in immunoprecipitates of both Parkin and p53 under hypoxic conditions (Figure 6C). An interaction between endogenous p53 and Parkin proteins was also found in hypoxic MCF7 cells (Figure 6D). These results suggest that p53 may interact with Parkin in the cytoplasm and disturb its translocation to the mitochondria, thereby blocking the process of mitophagy.

Re-expression of Parkin in TAT-ODD-p53 treated MDA-MB-157 cells restored radioresistance under hypoxic conditions. Moreover, inhibition of mitophagy by ATG7 knockdown reversed Parkin-induced radioresistance in TAT-ODD-p53 treated MDA-MB-157 cells under hypoxic conditions (Figure 6E). These results suggested that TAT-ODD-p53 regulates tumor cell radiosensitivity at least partly by inhibiting Parkin-mediated mitophagy.

## DISCUSSION

Resistance of many solid tumors to anticancer therapies is an important clinical problem and is partly determined by hypoxia of the tumor microenvironment. Previous studies have demonstrated that hypoxic cells in the tumor mass play a vital role in radioresistance, which means that killing hypoxic tumor cells is important in the treatment of solid tumors. The tumor suppressor p53 shows mutation or silencing in many tumor cells. After loss of p53 function, cancer cells escape from commitment to apoptosis and continue through the cell cycle without being arrested at cell cycle checkpoints [27]. The result of these alterations is generation of radioresistant tumor cells. We previously constructed a p53 peptide fused with TAT-ODD, which was proven to function efficiently in hypoxic cells and in solid tumors (colorectal adenocarcinoma and non-small lung cancer) [19, 20]. In the present study, we demonstrated that this TAT-ODD-P53 fusion protein was taken up by hypoxic breast cancer cells and was selectively stable in these cells. Moreover, treatment with TAT-ODD-P53 significantly enhanced the radiosensitivity of hypoxic breast cancer cells both *in vitro* and *in vivo*.

Mitophagy plays a key role in the removal of damaged mitochondria [28], and is an adaptive response that is reported to be induced by hypoxia and photo-irradiation [10, 29]. However, the role of mitophagy in radioresistance of hypoxic breast cancer cells has been unclear. Our present results demonstrated that mitophagy was markedly increased by hypoxia, leading to radioresistance of breast cancer cells. Parkin is an E3 ubiquitin ligase that has been reported to initiate mitophagy, which eliminated dysfunctional mitochondria and contributed to stress adaptation in a chemical hypoxia model. This study demonstrated that induction of mitophagy by hypoxia could be inhibited by the deletion of Parkin, suggesting the involvement of Parkin-mediated mitophagy in radioresistance of breast cancer cells under hypoxic conditions.

It is known that p53 not only regulates apoptosis, but also autophagy. It was reported that p53 promotes mitochondrial dysfunction by inhibiting Parkin-mediated mitophagy in mouse heart cells and pancreatic β-cells [17, 18]. However, there have been few studies on the role of p53 in cancer cell mitophagy. In the present study, we focused on the role of p53 in regulating mitophagy. Overexpression of exogenous p53 was demonstrated to repress the induction of mitophagy in irradiated hypoxic breast cancer cells, and this effect was reversed by overexpression of Parkin, while suppression of endogenous p53 had the opposite effect. However, p53 knockdown did not enhance mitophagy in Parkin-depleted hypoxic breast cancer cells. These results indicate that Parkin is required for the regulation of mitophagy by p53 in hypoxic breast cancer cells. Moreover, the radio-sensitizing effect of synthetic p53 peptides on hypoxic cells was weaker after autophagy was blocked by ATG7. In addition, overexpression of Parkin only reduced the radio-sensitizing effect of TAT-ODD-P53 in hypoxic cells not transfected with ATG7 siRNA. All of these findings support the hypothesis that p53 sensitizes hypoxic cancer cells to radiation by inhibiting Parkin-mediated mitophagy.

Parkin has been identified as a target gene of p53 and was reported to be a repressor of p53 transcription in neurons [30, 31]. However, this study revealed that p53 did not alter Parkin expression in hypoxic breast cancer cells, but instead affected translocation of Parkin to the mitochondria. While the role of p53 as a transcription factor in the activation of numerous genes has been studied extensively and new target genes are regularly reported, p53 also operates in the cytosol to modulate cell death and autophagy in a transcription-independent manner [14, 32]. This was also seen in the present study with regard to regulation of Parkin by p53. The Parkin-p53 complex was observed by immunoprecipitation of both Parkin and p53 (exogenous or endogenous) in the cytosolic lysate of hypoxic breast cancer cells, proving that a direct interaction occurred between these two proteins under hypoxic conditions. These results also support the previous report that p53 can either induce or inhibit autophagy in response to stress depending on its subcellular localization, and that cytoplasmic p53 suppresses the induction of autophagy [14].

In conclusion, TAT-ODD-p53 demonstrated a significant radiosensitizing effect on hypoxic solid tumor cells both *in vitro* and *in vivo*, suggesting that it has the potential to improve the therapeutic index of radiation therapy. Parkin-mediated mitophagy may contribute to the hypoxic radioresistance of breast cancer cells, and is regulated by a direct interaction between p53 and Parkin. Demonstration of a direct molecular link between activation of p53 and suppression of Parkin-mediated mitophagy has established a new mechanism of radiosensitization.

## MATERIALS AND METHODS

### Cell lines and culture

The MCF-7 human breast cancer cell line (wild-type p53) was obtained from the Cell Bank of Type Culture Collection of the Chinese Academy of Sciences (Shanghai, China). The MDA-MB-157 cell line (null p53) was obtained from the Cell Resource Center of Peking Union (Beijing, China). MCF-7 cells were maintained in DMEM (Gibco) supplemented with 10% FBS (Gibco). MDA-MB-157 cells were maintained in DMEM (Gibco) containing 10% FBS (Gibco), 1 mM glutamine, 1 mM sodium pyruvate, and 1% non-essential amino acids. All of the cells were cultured at 37°C under 5% CO_2_ in air.

### Peptide synthesis

Expression plasmids for TAT-ODD-p53, TAT-p53, TAT-ODD-EGFP, and p53 were constructed. Protein expression and purification were carried out as described previously [19]. All of the proteins were stored in 50 mM PBS at −80°C and were used within six months.

### Hypoxia and irradiation

Cells were subjected to hypoxia in a Forma 1029 anaerobic chamber (Thermo Fisher Scientific; Waltham, MA, USA) with a humidified atmosphere of 0.5% O_2_ and 5% CO_2_ balanced with N_2_ at 37°C. The cells were cultured under hypoxic conditions for 8 h before either being treated with synthetic p53 peptides or irradiated. Irradiation was done at a dose rate of 5.0 Gy/min using a 6-MV linear accelerator (LINAC; 2300EX; Varian Co., Palo Alto, CA, USA) at a distance of 100 cm from the source to the axis.

### Plasmids and RNA interference

p53 siRNA, Parkin siRNA, ATG7 siRNA and the plasmid encoding Parkin were obtained from Genepharma (Shanghai, China). Transfection was performed using Lipofectamine 2000 reagent (Invitrogen) according to the instructions provided by the manufacturer.

### Cytotoxicity assay

The cytotoxicity of synthetic p53 peptides for MCF-7 and MDA-MB-157 cells was studied to determine the concentrations for use in subsequent studies. Cells were plated into 96-well plates in 0.1 mL of medium and incubated overnight. On the following day, the cells were exposed to p53 peptide dissolved in PBS (0, 2, 4, 8, 16, or 32 μg/ml) for 72 h under normoxic or hypoxic conditions. Then 20 ml of 5 mg/mL MTT solution in PBS was added to each well for 4 h at 37°C. Subsequently, 20% sodium dodecyl sulfate (SDS) solution in 0.01% HCl (150 μL) was added to each well, before measurement at an absorbance of 570 nm using a spectrophotometric plate reader (ND-1000, Wilmington, DE). Two independent experiments were performed, with each being done in triplicate. The inhibitory concentration (IC) was determined for each peptide with each cell line.

### Colony-forming assay

The colony-forming assay was performed to determine radiosensitivity. Cells were plated in 6-well plates and allowed to adhere overnight before exposure to radiation at the indicated doses and a dose rate of 5 Gy/min using 6-MV X-rays generated by a linear accelerator (Varian 2300EX, Varian, Palo Alto, CA). After incubation for 10–14 days, the cells were stained with 0.5% crystal violet in methanol. Then the colonies (clusters of > 50 cells) were counted under a microscope. Survival data from different experiments were pooled, after which survival curves were fitted and analyzed by using the linear-quadratic model (LQ). The sensitizing enhancement ratio (SER) and oxygen enhancement ration (OER) were calculated as described previously [22, 33].

### Quantitative RT-PCR

Total RNA was extracted from cells using TRIzol reagent (Invitrogen) according to the manufacturer's protocol. Then reverse transcription was performed with the PrimeScript^®^ RT reagent kit (Takara), followed by real-time PCR using an ABI 7500 Sequence Detection System with a SYBR^®^ Premix Ex Taq™ kit (Takara). The sequences of the specific PCR primers were as follows: Parkin: Forward: 5′-GAGTCCAGGAGCTTGACACGAGT-3′, Reverse: 5′-AAGGGATGCTGCGCCTGTTGC-3′; p21: Forward: 5′-ATGTCCAATCCTGGTGATGT-3′, Reverse: 5′-TGCAGCAGGGCAGAGGAAGT-3′;β-actin: Forward: 5′-TCGACAACGGCTCCGGCAT-3′ Reverse: 5′-AAGGTGTGGTGCCAGATTTTC-3′. β-actin was used as the internal control.

### mtDNA assay

Total cellular DNA was extracted with a Universal Genomic DNA Extraction Kit (Takara) according to the manufacturer's protocol. Then real-time-PCR was performed using the PrimeScript ^®^ RT reagent kit (TaKaRa) and an ABI 7500 Sequence Detection System. The sequences of the primers were as follows: mt-ATP6 Forward: 5′-CGCCACCCTAGCAATATCAA-3′, Reverse: 5′-TTAAGGCGACAGCGATTTCT-3′; Rpl13: Forward: 5′-CTCGAGTCATCACTGAGGAA-3′, Reverse: 5′-CAACATCCTGTTCTGCGGCTT-3′. The level of gene expression was normalized by a standard curve and relative expression was calculated as mt-Atp6/Rpl13.

### Western blot analysis

Whole cell lysates were harvested and western blotting was conducted as described previously [34]. Mitochondrial and cytosolic fractions were isolated using a Qproteome™ Mitochondria Isolation kit (Qiagen) according to the manufacturer's instructions. Primary antibodies included rabbit anti-cleaved caspase-3 (Abcam), anti-p53 (Cell Signaling), anti-Parkin (Cell Signaling), anti-ATG7 (Cell Signaling) anti-COX IV, and anti-β-actin (Cell Signaling). Blots were visualized using an ECL detection kit (Millipore). Densitometric quantification of signal intensities was done with Image Pro Plus 6.0 software.

### Flow cytometric analysis of apoptosis

FITC-conjugated annexin V was used to detect apoptosis. Cells were seeded in 6-cm dishes and grown to 80% confluence. After irradiation, the cells were incubated for 24 h, followed by harvesting and staining with annexin V-FITC and PI (Invitrogen) according to the manufacturer's instructions. Then fluorescence was detected by flow cytometry.

### Tumor model

For the *in vivo* experiments, 1 × 10^7^ MDA-MB-157 cells were subcutaneously injected into the breasts of 6 to 8-week-old female Balb/c immunodeficient mice, as described previously [34]. To assess the distribution of p53 fusion protein and its stability in tumor tissues, each p53 fusion protein (1 mg/kg) or PBS was injected i.p. daily for five days into adult mice with established tumors approximately 200 mm^3^ in size (*n* = 3 per group). To visualize viable hypoxic cells, animals were injected with pimonidazole (60 mg/kg I.P. (hypoxyprobe-1, USA) at 60 min prior to being killed. Animal handling and these experiments were performed in accordance with the animal care guidelines of the Southern Medical University (Guangzhou, China).

### Assessment of tumor growth inhibition

Inhibition of tumor growth was studied in mice with tumors approximately 200 mm^3^ in size by injecting animals i.p. for five days with each p53 fusion protein (1 mg/kg) or PBS (*n* = 5 per group). A single dose of radiation (10 Gy) was given on day 5. Tumor dimensions were measured with calipers and the volume was calculated by using the formula V = (*a* × b^2^)/2, in which a and b are the largest and smallest perpendicular diameters, respectively. Time zero was the same for all four treatment conditions.

### TUNEL assay for apoptosis

The terminal deoxynucleotidyl transferase UTP nick end-labeling (TUNEL) assay was performed on frozen tumor tissue sections at seven days after exposure, using an In Situ Cell Death Detection Kit (Roche Diagnostics, 12156792910, Branford, CT, USA) according to the manufacturer's instructions. After washing 3 times for 5 min each in PBS, the sections were mounted in fluorescence mounting medium with DAPI (Invitrogen) to identify the nuclei.

All of the paired sections were examined under a fluorescence microscope (Olympus, Japan).

### Preparation of stable GFP-LC3-expressing cells

A recombinant lentivirus containing GFP-LC3 was obtained from Genepharma (Shanghai, China). MDA-MB-157 cells and MCF7 cells were infected with lentivirus particles and isolated by fluorescence-activated cell sorting to obtain t cells that stably expressed GFP-LC3.

### Immunocytochemistry and confocal microscopy

A total of 5 × 10^4^ cells were plated overnight in a confocal dish (Corning, USA). Then the cells were cultured under hypoxic (0.5% O_2_) or normoxic (20% O_2_) conditions for 8 h before irradiation. After irradiation (6 Gy), the cells s were loaded with 200 nM MitoTracker Red (Molecular Probes, M7512) by culture for another 6 h at 37°C before being fixed for 30 minutes with 4% paraformaldehyde. Subsequently, the cells were mounted with DAPI mounting medium (Invitrogen) and observed under a fluorescence confocal microscope (Olympus FV10i) for confocal imaging using Olympus FV10-ASW software.

### Transmission electron microscopy

A total of 5 × 10^5^ cells were seeded in 6-cm dishes overnight and then were cultured under hypoxic (0.5% O_2_) conditions for 8 h. After irradiation (6 Gy), the cells were cultured for 6 h before harvesting, and were kept overnight in 2% paraformaldehyde and 2.5% glutaraldehyde in 0.1 M PBS (pH 7.4) before being cut into 50-μm sections on a vibratome. Selected areas were processed by post-fixing in 1% osmium tetroxide for 1 h, followed by dehydration in a graded ethanol series and embedding in epoxy resin. Polymerization was performed for 24 h at 80°C. Ultrathin sections (100 nm) were cut, stained with uranyl acetate and lead citrate, and viewed under an AMTTEM camera system (Hitachi-H7500).

### Co-Immunoprecipitation assay

Proteins were extracted from the cytoplasmic fraction using a PARIS kit (Ambion) according to the manufacturer's instructions. Co-immunoprecipitation was performed using the Pierce Co-Immunoprecipitation Kit (26149, Pierce; Rockford, IL) by following the manufacturer's instructions. The lysates were applied to columns containing 10 μg of immobilized antibodies (p53 or Parkin) covalently linked to an amine-active resin and incubated overnight at 4°C. Then the co-immunoprecipitate was eluted and analyzed by SDS-PAGE along with the controls.

### Statistics

Unless stated otherwise, all of the experiments were conducted in triplicate. Data were analyzed for statistical significance by using Student's *t*-test or one-way ANOVA. Results are presented as the mean ± standard deviation, and a *P* value < 0.05 was considered to indicate statistical significance.
